# The role of the Coronavirus pandemic on childbearing intentions in Iranian women based on path analysis: A cross-sectional study

**DOI:** 10.18502/ijrm.v21i1.12665

**Published:** 2023-02-08

**Authors:** Mojdeh Banaei, Nourossadat Kariman, Hamid Sharif Nia, Tahereh Mokhtarian-Gilani

**Affiliations:** ^1^Mother and Child Welfare Research Center, Hormozgan University of Medical Sciences, Bandar Abbas, Iran.; ^2^Midwifery and Reproductive Health Research Center, School of Nursing and Midwifery, Shahid Beheshti University of Medical Sciences, Tehran, Iran.; ^3^Traditional and Complementary Medicine Research Center, Addiction Institute, Amol Faculty of Nursing and Midwifery, Mazandaran University of Medical Sciences, Sari, Iran.; ^4^Student Research Committee, School of Nursing and Midwifery, Shahid Beheshti University of Medical Sciences, Tehran, Iran.

**Keywords:** Iran, Delayed childbearing, Reproductive behavior, COVID-19, Attitude.

## Abstract

**Background:**

The Coronavirus disease (COVID-19) may lead to couples not being physically and mentally ready to assume a parenting role.

**Objective:**

Given the changes in reproductive behaviors and the lack of accurate information about childbearing factors during the Coronavirus pandemic, this study was conducted to investigate the role of the COVID-19 pandemic in Iranian couples' childbearing intentions based on the theory of planned behavior model.

**Materials and Methods:**

This cross-sectional study was conducted on 400 married Iranian women from July to October 2020 using official online popular social networks. Data were collected using a demographic checklist and the researcher-made questionnaire, which was designed based on the main constructs of the planned behavior model.

**Results:**

Testing the indirect relationships of the mediation model effect showed a positive relationship between knowledge (β = 0.226, p < 0.001) and subjective norms (β = 0.155, p = 0.001) about COVID-19. Anxiety about COVID-19 mediated the relationship of knowledge (β = 0.105, p = 0.009), attitude (β = -0.125, p = 0.002), subjective norms (β = 0.238, p < 0.001), and perceived behavioral control (β = 0.513, p < 0.001) about COVID-19 with childbearing intentions.

**Conclusion:**

The results showed that COVID-19-induced anxiety can affect the relationship between the components of the theory of planned behavior model and childbearing intentions. Therefore, it is suggested that by designing appropriate interventions through anxiety-reducing and relaxation techniques, a fundamental step can be taken in increasing childbearing desires.

## 1. Introduction

The novel Coronavirus disease (COVID-19) is a newly-emerged global challenge that has rapidly spread worldwide (1). As the frequency of infections and deaths by COVID-19 rises, people experienced more mental problems such as depression, stress, and anxiety (2). Moreover, given that most couples of reproductive ages are likely to get pregnant, they inevitably have to decide about the right time to have children (3). The COVID-19 pandemic may make couples physically and mentally unprepared to assume a parenting role, since there is a limited range of information about this disease (4), and the information limitations in this area exacerbate people's fears and concerns affecting their childbearing intentions (5).

Although, some studies have suggested that women's likelihood of infection with the Coronavirus increases during pregnancy, and they could also transmit the virus to their fetus (6); however, some studies have also reported the possibility of testicular damage and infertility in men who contract COVID-19 (7, 8). These findings can make it difficult for couples to choose the right time for childbearing. A study conducted in Italy showed that the COVID-19 pandemic affects childbearing intentions, but did not refer to the factors predicting childbearing intentions during this period (5). Therefore, since reproduction is a voluntary behavior that is affected by many factors, it is better to investigate these factors through behavior change theories for a more accurate explanation of this behavior (9). One of the theories of interest to demographics in this area is the theory of planned behavior (TPB). The TPB is the most appropriate and complete theory for studying behavior (10). Caplescu used the TPB to study fertility intentions in Romania. The results indicate that age, the number of children, woman's attitude and perception of her significant others' attitude toward having a birth in future are important in determining the odds that a woman will intend to decide for childbearing (11).

One of the TPB's main opportunities is assessing change in a person's attitude, norms, and intention over time and in particular circumstances, including the COVID-19 pandemic (12). Based on the evidence, the lack of knowledge mainly leads to a negative attitude, which may affect the intention to perform a behavior. These epidemics and outbreaks often have severe consequences and may even affect people's mental health (13).

Consequently, given the changes in reproductive behaviors and the lack of accurate information about the childbearing factors during the Coronavirus pandemic, the present study was carried out to examine the role of the COVID-19 pandemic in Iranian couples' childbearing intentions based on the planned behavior model. While providing an overview of the current situation, this study seeks to facilitate policymakers' decision-making based on the existing laws by identifying the factors related to this decision and through appropriate interventions designed by health service authorities and providers.

## 2. Materials and Methods

### Participants, recruitment setting, and sampling procedure

The present cross-sectional study was conducted on 400 married Iranian women from July to September 2020. The sample size was determined with a 95% confidence interval, 5% error, 33% prevalence (14), and 10% withdrawals.

Initially, married Iranian women of reproductive age (15-45 yr) with reading and writing literacy were included in the study, whereas pregnant, breastfeeding, postmenopausal, and infertile women were excluded. Given the COVID-19 pandemic, the study was conducted online using popular official social networks among the public such as WhatsApp, Instagram and etc. The research poster was published on social networks to inform the public and contained a brief introduction to the research history, the study objectives, participants' characteristics, the voluntary nature of participation in the study, the confidentiality of the data, and a link to the online questionnaire. The eligible participants willing to participate in the study first completed the informed consent form at the top of the electronic questionnaire and then completed the questionnaires.

### Measures

Data were collected using the demographic questionnaire and the childbearing intentions and related factors questionnaire.

### Social and demographic variables

The demographic questionnaire was researcher-made and inquired about age, spouse's age, duration of the marriage, education, occupation, monthly household income, housing status, and household size.

### Childbearing decision-making factors

The researcher-made “factors related to childbearing intentions during the COVID-19 pandemic” questionnaire was designed according to the main constructs of the TPB (15) and literature review, and was validated using face and content validity methods. This questionnaire asked about the effect of the COVID-19 pandemic on childbearing intentions, the number of children, appropriate birth spacing, and the right time to have children. To assess its face validity, the questionnaire was distributed among 15 eligible women, who were asked to comment on its appearance, clarity of the chosen words, and the logical sequence of the items. The impact score was also calculated to examine the quantitative face validity, and the item was kept for the next analyses if the impact score was > 1.5 (impact score of all items was > 1.5 and acceptable). To assess validity, 8 experts (reproductive health experts, midwives, and epidemiologists) were invited, and the content validity ratio and content validity index were determined (content validity ratio = 0.750, I-content validity index = 0.875, Kappa statistic ${}^{*}$ = 0.870). The reliability of the questionnaire's dimensions was assessed using the internal consistency method with Cronbach's alpha and Kuder Richardson coefficients. The “factors related to childbearing intentions during the COVID-19 pandemic” questionnaire contained 33 items in 4 dimensions, as follows:

#### Knowledge and attitude about COVID-19

This dimension included 16 items related to knowledge and 3 related to the attitude of couples about the decision to have children during the COVID-19 pandemic. “False” or “I don't know” responses scored zero in the knowledge part, correct answers scored one point, and the scoring ranged from 0-16 in this part. The responses to the attitude questions scored one point for “agree,” 2 for “disagree,” and 3 for “no comments,” and the scores for this part ranged from 3-9. The reliability of this dimension was assessed with Kuder Richardson coefficient of 0.611 for the knowledge questions and Cronbach's alpha coefficient of 0.70 for the attitude part.

#### Subjective norms about COVID-19

This dimension of the questionnaire was assessed with 4 questions: 1) People think we should have children as soon as possible; 2) I think others will ridicule me if I have children; 3) Physicians (midwives) advise against childbearing during the Coronavirus spread; and 4) My physician (midwife) thinks that I should have children sooner rather than later because of my fertility age limit. The scores in this dimension ranged from 1 for “totally disagree” to 7 for “totally agree,” with the minimum score of 4 for the perceived subjective norms, and a maximum of 28. The reliability of this dimension was determined with Cronbach's alpha coefficient of 0.60.

#### Perceived behavioral control toward COVID-19

This dimension was assessed with 4 questions: 1) I'm going to have children even if it is too expensive; 2) I shall not be deprived of the blessing of having children because of the Coronavirus problems and barriers; 3) I can look after myself and my child; and 4) Financial problems, especially after the spread of the Coronavirus, do not let me consider having children. The scores ranged from 1 for “totally disagree” to 7 for “totally agree,” with the minimum score of 4 and a maximum of 28 for perceived behavioral control. The reliability of this dimension was determined with Cronbach's alpha coefficient of 0.83.

#### Anxiety about COVID-19

This dimension included 6 items and the answers to the questions ranged from 1 point for “totally disagree” to 7 for “totally agree”: 1) I am extremely worried about the spread of the Coronavirus; 2) I believe I might catch the Coronavirus at any given moment; 3) I'm concerned about transmitting the Coronavirus to those around me; 4) My daily activities have been disrupted by Coronavirus-related anxiety; 5) I'm extremely worried by thoughts of getting pregnant and visiting for prenatal care and tests during the Coronavirus outbreak; and 6) Thinking about going to the hospital for childbirth during the Coronavirus outbreak makes me extremely anxious. The scores in this dimension ranged from 6-42. The reliability of this dimension was determined with Cronbach's alpha coefficient of 0.80.

### Ethical considerations 

This research was approved by the Ethics Committee of the Research Deputy of Shahid Beheshti University of Medical Sciences, Teheran, Iran (Code: IR.SBMU.RETECH.REC.1399.390). Sampling began after obtaining the necessary permissions from the university authorities. The purpose of this study was explained to the participants. The eligible participants confirmed the study consent form at the beginning of the electronic questionnaire and, then, completed the questionnaires online. The phone number of the researcher was provided to the participants, and they were assured that all of their responses would be kept confidential.

### Statistical analysis

Statistical Package for the Social Sciences, version 26.0, SPSS Inc, Chicago, Illinois, USA. was used to summarize the demographic characteristics of the subjects. Categorical variables and continuous variables were summarized using frequencies, percentages, arithmetic mean, and standard deviation, respectively. The missing data were replaced using the mean imputation method. Pearson's correlation analysis assessed the relationship between the study variables (i.e., knowledge, attitude, subjective norms, perceived behavioral control, and anxiety about COVID-19) and childbearing intentions. The present research used PROCESS macro to explore the research mediation model. First, the direct relationships between the 4 concepts including knowledge, attitude, subjective norms, and perceived behavioral control about COVID-19 and childbearing intentions, were tested without including anxiety about COVID-19 (total effects model). Second, the mediator (i.e., anxiety about COVID-19) was added to the model to develop a mediation model (mediation effects model).

Knowledge, attitude, subjective norms, and perceived behavioral control about COVID-19 were modeled as antecedent variables influencing childbearing intentions directly and indirectly through anxiety about COVID-19 as the mediator. The model was assessed using Analysis of a Moment Structures Software (AMOS), version 24.0, PA 15090 USA. All the path coefficients were estimated using the maximum likelihood method, and their significance was assessed using bootstrapping with 2000 replications. Next, the bootstrapping approach estimated the standard error of the indirect relationships. Bootstrapping is more accurate and has higher statistical power than the approaches proposed. The coefficient of determination (R^2^) was also calculated to evaluate how well the model explained the outcome.

## 3. Results 

### Sample characteristics

The mean age of the participants was 33.41 ± 5.36 yr, and the mean age of their partners was 35.38 ± 5.30 yr. The mean duration of marriage was 9.53 ± 5.65 yr, and the mean parity was 1.04 ± 0.83. Table I presents other demographic details and the effect of COVID-19 on the timing of childbearing, birth spacing, and the desired number of children in participants' views. In the present study, only 40.3% of the participants were willing to have children during the COVID-19 pandemic.

### Correlation 

Table II shows that knowledge (r = 0.129, p = 0.01), subjective norms (r = 0.244, p < 0.001), and perceived behavioral control (r = 0.579, p < 0.001) about COVID-19 had a significant positive relationship with childbearing intentions. Anxiety (r = -0.127, p = 0.01) and attitude (r = -0.303, p < 0.001) about COVID-19 were negatively correlated with childbearing intentions.

Assessing the total effects mediation model showed a significant relationship for knowledge (β = 0.084, p = 0.03), attitude (β = -0.132, p < 0.001), subjective norms (β = 0.223, p < 0.001), and perceived behavioral control (β = 0.527, p < 0.001) about COVID-19 with childbearing intention.

### Path analysis model

Table III confirms that the mediation model examines the relationship of knowledge, attitude, subjective norms, and perceived behavioral control about COVID-19 with childbearing intentions. Nonetheless, does not have a relationship between attitude and anxiety about COVID-19 (β = 0.071, p = 0.170).

Testing the indirect relationships of the mediation model showed a positive relationship between knowledge (β = 0.226, p < 0.001) and subjective norms (β = 0.155, p = 0.001) about COVID-19. Also, a negative relationship was found between anxiety and perceived behavioral control (β = -0.146, p = 0.001) about COVID-19 with childbearing intentions (β = -0.096, p = 0.01).

**Figure 1:**
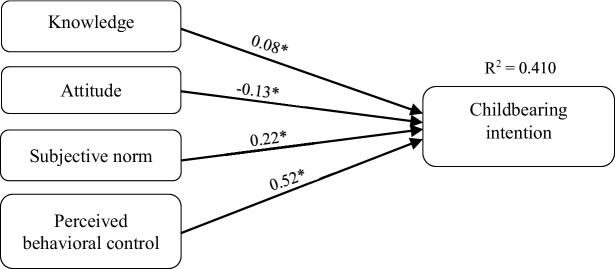
The total effects model of childbearing intentions based on the TPB during the Coronavirus pandemic.

**Figure 2:**
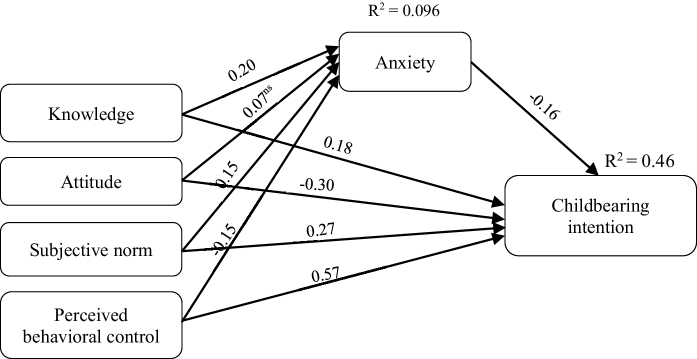
The total effects and mediation effects models of childbearing intentions based on the TPB during the Coronavirus pandemic.

Anxiety about COVID-19 mediated the relationship of knowledge (β = 0.105, p = 0.009), attitude (β = -0.125, p = 0.002), subjective norms (β = 0.238, p < 0.001), and perceived behavioral control (β = 0.513, p < 0.001) about COVID-19 with childbearing intentions. The model explained 41% of the variance in anxiety and 46% of the variance in childbearing intentions (Figures 1 and 2).

**Table 1:** The demographic profiles of the respondents


**Variable**		**Frequency (percentage)**
**Educational status**		
	**Elementary school**	5 (1.3)
	**Secondary school**	8 (2)
	**High school**	86 (21.5)
	**Diploma and university**	301 (75.2)
**Socio-economic status**		
	**Lower income**	29 (7.3)
	**Middle income**	203 (50.7)
	**Higher income**	168 (42)
**Employment status**		
	**Housewife**	197 (49.3)
	**Employee**	203 (50.7)
**Housing status**		
	**Mortgage**	25 (6.3)
	**Rent**	110 (27.5)
	**Owner**	227 (56.7)
	**Live with family**	28 (7)
	**Organization**	10 (2.5)
**COVID-19 effect on the childbearing intention**		
	**Yes**	161 (40.3)
	**No**	239 (59.7)
**COVID-19 effect on the timing of childbearing**		
	**Yes**	174 (43.5)
	**No**	226 (56.5)
**COVID-19 effect on childbearing spacing**		
	**Yes**	150 (37.5)
	**No**	250 (62.5)
**COVID-19 effect on your number of childbearing**		
	**Yes**	99 (24.8)
	**No**	301 (75.2)
COVID-19: Coronavirus disease		

**Table 2:** The results of the correlation analysis


	**Mean ± SD**	**Attitude**	**Subjective norm**	**Perceived behavioral control**	**Anxiety**	**Childbearing intention**
**Knowledge**	10.60 ** ± ** 2.18	0.257 ${}^{**}$	-0.005 * ${}^{ns}$ *	0.023 * ${}^{ns}$ *	0.204 ${}^{**}$	0.129 ${}^{*}$
**Attitude**	5.76 ** ± ** 1.72	0.035 * ${}^{ns}$ *	-0.297 ${}^{**}$	0.062 * ${}^{ns}$ *	-0.303 ${}^{**}$
**Subjective norm**	14.80 ** ± ** 3.40		-0.047 * ${}^{ns}$ *	0.150 ${}^{**}$	0.244 ${}^{**}$
**Perceived behavioral control**	19.12 ** ± ** 3.48		-0.155 ${}^{**}$	0.579 ${}^{**}$
**Anxiety**	30.19 ** ± ** 8.41			-0.127 ${}^{*}$
**Childbearing intention**	4.45 ± 2.48			
${}^{ns}$ P ≥ 0.05, ${}^{*}$ P < 0.05, ${}^{**}$ P < 0.01, two-tailed tests						

**Table 3:** The direct, indirect, and total effects


	**Standardized path**	**95% Confidence interval**	
	**Path**	coefficients ${}^{*}$	**P-value**	**Lower bound**	**Upper bound**
**Total effects**					
	**Knowledge → Childbearing intention**	0.084	0.036	0.006	0.184
	**Attitude → Childbearing intention**	-0.132	0.001	-0.310	-0.072
	**Subjective norm → Childbearing intention**	0.223	< 0.001	0.107	0.218
	**Perceived behavioral control → Childbearing intention**	0.527	< 0.001	0.318	0.431
**Indirect effects**					
	**Knowledge → Anxiety**	0.226	< 0.001	0.496	1.246
	**Attitude → Anxiety**	0.071	0.170	-0.150	0.849
	**Subjective norm → Anxiety**	0.155	0.001	0.151	0.617
	**Perceived behavioral control → Anxiety**	-0.146	0.003	-0.592	-0.115
	**Anxiety → Childbearing intention**	-0.096	0.017	-0.051	0.005
**Direct effects**					
	**Knowledge → Childbearing intention**	0.105	0.009	0.029	0.211
	**Attitude → Childbearing intention**	-0.125	0.002	-0.299	0.062
	**Subjective norm → Childbearing intention**	0.238	< 0.001	0.118	0.229
	**Perceived behavioral control → Childbearing intention**	0.513	< 0.001	0.307	0.422
${}^{*}$ Pearson's correlation analysis					

## 4. Discussion 

This study evaluated the mediating role of anxiety about COVID-19 in the relationship between childbearing intentions and TPB dimensions in Iranian women.

According to recent evidence, psychosocial factors in TPB are effective in the formation of fertility goals (11). In the present study, the desire to have children had a significant relationship with constructs including knowledge, attitude, subjective norms, perceived behavioral control during COVID-19, and anxiety about COVID-19. Similarly, another study also found that all the constructs of TPB affected childbearing intentions, while perceived behavioral control was the strongest construct determining the desire to have children, followed by attitude, and subjective norms (16). In another study, the researchers concluded that childbearing intentions have positive and significant relationships with all TPB constructs (attitude, subjective norms, and perceived behavioral control) (17). In general, childbearing intentions are commonly known as direct predecessors of reproductive behaviors which are defined as conscious and psychological states that mediate between various factors and the behavioral outcome (15, 18). On the other hand, the impact of COVID-19 pandemic on the daily life of millions of people is unprecedented, and its long-term consequences are still unclear (19). People's reaction to changing conditions such as current pandemic is affected on their fertility and childbearing intentions (20).

Various studies have demonstrated the effect of attitude on fertility goals (21, 22). Attitude means “The degree of an individual's favorable or unfavorable assessment of the intended behavior.” In other words, attitude perceives and classifies the individual perspectives, beliefs, and feelings toward a broad range of different issues (23). In the population examined in the present study, 17.3% had negative attitudes, 49.6% had no comments, and 37.9% had positive attitudes toward the intended behavior (i.e., childbearing during the pandemic). Positive and negative attitudes affected childbearing desire (24). A study reported that anxiety and concerns about pregnancy and childbearing increased during the COVID-19 pandemic (25). In another study, it was shown that women's fear of limited access to doctors was much greater than the fear of being infected with COVID-19 because they thought that they might be alone during labor or after childbirth due to the absence of a doctor in the hospital (20). In addition, some women may decide to delay a pregnancy because they are afraid that they or their close ones might get sick (26). Therefore, several psychological factors, such as fear, anxiety, and stress, are likely to affect the individual's attitude (13). A study concluded that the major attitudes toward COVID-19 (i.e., “totally agree” or “totally disagree”) could easily be changed through anxiety and stress (27). The anxiety and concerns among the public largely vary during the COVID-19 pandemic. Recent evidence suggests that people in quarantine have significant levels of anxiety, stress, and anger (13).

According to the present findings, COVID-19-induced anxiety had significant relationships with the scores of knowledges, subjective norms, perceived behavioral control, and childbearing desire. In agreement with the present findings, a study showed that the concern and anxiety induced by the COVID-19 pandemic impacted the desire of couples who had already planned to have children. In addition, most study subjects who had planned to have children before the Coronavirus outbreak belonged to older age groups and were, therefore, more anxious and concerned about future infertility than the consequences of infection with the virus (5). Given these contexts, the negative impact of the pandemic on fertility programs should not be underestimated. In contrast to the present study, another study showed no significant relationship between the score of knowledge and COVID-19-induced anxiety (28). In another study, COVID-19 knowledge had negative associations with symptoms of general anxiety, depression, and psychological distress (29). Moreover, according to recent evidence, in the event of phenomena with high-mortality rates, following an initial decline in fertility, fear and anxiety about losing one's child became a further motivation to have more children (30). In the present study, the higher scores of knowledge, subjective norms, and perceived behavioral control about COVID-19, directly and indirectly, led to an increase in childbearing intentions; however, the attitude toward COVID-19 only directly affected the desire to have children without the mediation of anxiety. As reported in previous epidemics, illness and quarantine may have a major effect on pregnancy and childbirth, but it is still not clear whether and how COVID-19 affects the rate of childbirth and if it reduces the rates or not (5).

In the present study, among the constructs of TPB, COVID-19-induced anxiety had a mediating role in the intention to carry out the behavior (childbearing desire). A recent study on the trend of changes in psychological symptoms during previous pandemics have shown that although the initial reactions to a pandemic are identified with increasing levels of anxiety and concern, these symptoms gradually abate in the pandemic (13). Therefore, interventions to increase childbearing desire through anxiety-reducing and relaxation techniques will be more effective. Holding web-based (virtual) classes and providing information about safety techniques against COVID-19 infection before and during pregnancy, during childbirth, and in the postpartum period will probably reduce some of the further stresses and anxieties experienced by couples. Women should routinely be screened for psychosocial vulnerabilities. In addition, there is no evidence suggesting that pregnant women are more prone to COVID-19 infections or more severe complications following infection with this virus (31). Consequently, because of the inadequate knowledge about COVID-19, couples should be more conservative in deciding to cancel their pregnancy plans. The COVID-19 pandemic provides an opportunity to reform existing policies and help the government to develop more effective measures to improve people's fertility intention to have children.

The strengths of the present study include using a path analysis model to test the TPB model and determining all the path coefficients using maximum likelihood with the bootstrapping approach. One of the limitations of the study was the electronic sampling because of the COVID-19 pandemic. Other limitations include the fact that there was no limitation on the couples' parity. However, decisions made about the first, second, and next children differ in nature. Although younger and older parents' decision to have more children may be somehow different from what it was in the past, the decision to have the first child is expected to have more widely changed. With the assumption that women have the most information on childbearing and are the ultimate decision-makers in this regard, the target group of this study included only women. The researchers recommend conducting further studies to determine the factors predicting childbearing decision-making in the COVID-19 pandemic in men and also jointly with women.

## 5. Conclusion

The results showed that COVID-19-induced anxiety can affect the relationship between the components of the TPB model (knowledge, subjective norms, and perceived behavioral control) and childbearing intentions. And if this anxiety is not controlled, it may lead to adverse consequences in the individual and society. So given that anxiety plays an essential role in the occurrence of this behavior, it is suggested that by designing appropriate interventions through anxiety-reducing and relaxation techniques, a fundamental step was taken in increasing childbearing desires.

##  Conflict of Interest

The authors have no conflict of interest relevant to this article.
